# Response of photosynthetic characteristics and yield of grape to different CO_2_ concentrations in a greenhouse

**DOI:** 10.3389/fpls.2024.1378749

**Published:** 2024-07-22

**Authors:** Yufan Zhou, Hossam Salah Mahmoud Ali, Jinshan Xi, Dongdong Yao, Huanhuan Zhang, Xujiao Li, Kun Yu, Fengyun Zhao

**Affiliations:** The Key Laboratory of Characteristics of Fruit and Vegetable Cultivation and Utilization of Germplasm Resources of the Xinjiang Production and Construction Corps, Shihezi University, Shihezi, China

**Keywords:** air injection system, CO_2_, grapes, photosynthesis, yield, subsurface drip

## Abstract

Due to the enclosed environment of greenhouse grape production, the supply of CO_2_ required for photosynthesis is often insufficient, leading to photosynthetic downregulation and reduced yield. Currently, the optimal CO_2_ concentration for grape production in greenhouses is unknown, and the precise control of actual CO_2_ levels remains a challenge. This study aims to investigate the effects of different CO_2_ concentrations on the photosynthetic characteristics and yield of grapes, to validate the feasibility of a CO_2_ gas irrigation system, and to identify the optimal CO_2_ concentration for greenhouse grape production. In this study, a CO_2_ gas irrigation system combining CO_2_ enrichment and gas irrigation techniques was used with a 5-year-old Eurasian grape variety (*Vitis vinifera* L.) ‘Flame Seedless.’ Four CO_2_ concentration treatments were applied: 500 ppm (500 ± 30 µmol·mol^−1^), 700 ppm (700 ± 30 µmol·mol^−1^), 850 ppm (850 ± 30 µmol·mol^−1^), and 1,000 ppm (1,000 ± 30 µmol·mol^−1^). As CO_2_ concentration increased, chlorophyll a, chlorophyll b, and carotenoids in grape leaves all reached maximum values at 700 ppm and 850 ppm during the same irrigation cycle, while the chlorophyll a/b ratio was lower than at other concentrations. The net photosynthetic rate (Pn) and water use efficiency (WUE) of grape leaves were the highest at 700 ppm. The transpiration rate and stomatal conductance at 700 ppm and 850 ppm were significantly lower than those at other concentrations. The light saturation point and apparent quantum efficiency reached their maximum at 850 ppm, followed by 700 ppm. Additionally, the maximum net photosynthetic rate, carboxylation efficiency, electron transport rate, and activities of SOD, CAT, POD, PPO, and RuBisCO at 700 ppm were significantly higher than at other concentrations, with the highest yield recorded at 14.54 t·hm^−2^. However, when the CO_2_ concentration reached 1,000 ppm, both photosynthesis and yield declined to varying degrees. Under the experimental conditions, the optimal CO_2_ concentration for greenhouse grape production was 700 ppm, with excessive CO_2_ levels gradually inhibiting photosynthesis and yield. The results provide a theoretical basis for the future application of CO_2_ fertilization and gas irrigation techniques in controlled greenhouse grape production.

## 1 Introduction

With the development of modern industry, the world sees an increasing consumption of coal, oil, natural gas, and fossil fuels, resulting in a sharp rise of CO_2_ concentration in the atmosphere from 278 ppm before the Industrial Revolution to 420.41 ppm at present, with an annual growth rate of 2 ppm. It is expected that the CO_2_ concentration will increase to 800 ppm by the end of the 21st century (Davis, 2023). Relevant studies suggest that changes in CO_2_ concentration have a direct effect on photosynthesis and further influence the final yield of plants (Rakhmankulova et al., 2023). Plants are cultivated more densely during greenhouse cultivation, making a smaller total photosynthetic area for every plant. Greenhouses are less ventilated and even closed all day due to heat preservation, moisture preservation, and other types of pressure. Failure to timely replenish the greenhouse with CO_2_ usually leads to a shortage in CO_2_, causing a severe adverse effect on the plants’ photosynthesis and yield formation even though CO_2_ concentrations in the atmosphere will increase significantly (Zhang et al., 2023b). Photosynthetic performance is one of the significant predictive indicators of plant yield formation. Researchers have conducted considerable research on this aspect. The final results show that the increase in CO_2_ concentration has a substantial effect on the improvement of plant photosynthetic performance and the increase of fruit yield under the condition of sufficient moisture and nutrients (Kimball, 2016; Wei et al., 2024). A moderate increase in CO_2_ concentrations in the environment can effectively improve the yield and quality of grapefruits (Kizildeniz et al., 2015). Nevertheless, no specific studies are available on the plant mechanism of photosynthetic response to different high CO_2_ concentrations in facility production. For this reason, we need to determine the CO_2_ concentration required for plants under facility conditions to achieve optimal photosynthetic performance. Meanwhile, an exploratory study on the changes in characteristic parameters of plant photosynthesis enables us to predict plant growth and yield under different atmosphere CO_2_ concentrations in the future and finally achieve a high plant yield and efficiency and sustainable agricultural development under controllable conditions in the facility.

Many studies have discovered the promotive effect of elevated CO_2_ concentration on plant photosynthesis. Growth chamber, closed-top chamber, open-top chamber (OTC), and free-air CO_2_ enrichment (FACE) experiments have been performed over the past three decades to simulate the response of plant growth to elevated CO_2_ concentration (Miyoshi et al., 2017; Wang et al., 2022). Due to the reduction of carbon constraint, elevated CO_2_ concentration can improve the photosynthetic efficiency of plants and enhance the supply of photoassimilates (Linkosalo et al., 2017). Moreover, elevated CO_2_ concentration helps improve the net Pn of plant leaves (Ariura et al., 2023). Such gain effect is a typical result where elevated CO_2_ concentration promotes the RuBisCO carboxylation reaction of plant leaves, inhibits the RuBisCO oxygenation reaction, and increases the total amount of carbon (C) fixation in the photosynthetic metabolic pathway (Suboktagin et al., 2023). Many studies have shown that elevated CO_2_ concentration can induce plant leaf stomatal closure, increase stomatal resistance, reduce stomatal conductance, and improve water use efficiency (Gamage et al., 2018; Zong et al., 2021). While affecting the photosynthetic efficiency of plants, elevated CO_2_ concentration significantly affects the characteristic parameter change of CO_2_ response and light response (Ofori-Amanfo et al., 2021). Therefore, determination of the plant photosynthesis–CO_2_ response curve is identified as an essential method to learn about the photosynthetic capacity of plants. Based on fitting analysis, we can estimate many important photosynthetic parameters, such as apparent quantum yield (AQY), light compensation point (LCP), light saturation point (LSP), maximum net photosynthetic rate (*P*_nmax_), CO_2_ saturation point (Cisat), and CO_2_ compensation point (CCP). Many studies have shown that elevated CO_2_ concentration effectively increases the *P*_nmax_, LSP, AQY, and Cisat of plants and reduces LCP and CCP (Tang et al., 2017; Hou et al., 2021). Under the CO_2_ concentration of 1,000 to 1,500 µmol·mol^−1^, tomato yield was increased by 38% (Shimomoto et al., 2017); under CO_2_ concentration of 700 µmol·mol^−1^, tomato yield was increased by 125% (Dorneles et al., 2019). Under the condition that CO_2_ concentration is 60 µmol·mol^−1^ higher than the environment, the yield of rice was increased by 11.4% to 19.7% compared with elevated CO_2_ concentration (Zheng et al., 2018). Some studies have found that elevated CO_2_ concentration can significantly affect the antioxidant enzyme activity of plants, which may be a potential cause of changes in fruit yield (Jo et al., 2022). Most researchers only study the response of plants to elevated CO_2_ concentration at a single high CO_2_ concentration level (for example, 550, 850, or 900 µmol·mol^−1^), thereby limiting the prediction of optimal CO_2_ concentrations for plants under facility conditions.

China is the world’s largest producer and consumer of table grapes, with a grape production of 14.998 million tons in 2021 and a domestic output value of approximately US $400 billion (Medda et al., 2022). Grapes cultivated in the open field are directly affected by climate change, resulting in many factors limiting grapes’ growth, yield, and quality. Moreover, the field conditions are very complex, and the cost of artificially increasing CO_2_ is too high, but the benefit is low (Vogel et al., 2019). The grapes in the facility environment are located in a closed space, and the concentration of CO_2_ can be precisely controllable to obtain the best yield (Ainsworth and Long, 2021). Therefore, there is a need to study the response of facility grapes to different CO_2_ concentrations, helping field-grown grapes adapt to and mitigate climate change and optimize the facility production system. With the continuous development and improvement of aerated irrigation technology in recent years, several studies have found that the short-term application of aerated irrigation technology significantly improves soil aeration and water use efficiency near plant roots, effectively mitigating the problem of plant root hypoxia and then increasing the fruit yield (Zhu et al., 2022; Jin et al., 2023). Aerated irrigation technology uses an intelligent central control system for scientific and accurate gas injection and irrigation on plants. Furthermore, aerated irrigation in relevant current studies mainly uses O_2_ injection into the roots (Ouyang et al., 2020). However, the type and concentration of gas sources are too simple, causing many limitations. In earlier studies, suspended chemical bags, canopy enrichments, and blow-in injections were employed by the CO_2_ enrichment technology, failing to control the gas concentration accurately. With the application of OTC and FACE systems for CO_2_ enrichment in recent years (Lewin et al., 2009; Choi et al., 2017), CO_2_ is directly injected into an enclosed or semi-enclosed space, and its concentration remains constant at a single level, which overlooks the effect of gas on the plant root–soil environment. Based on the benefits of CO_2_ fertilization, our laboratory uses CO_2_ as the source of gas injection, combines CO_2_ enrichment technology with aerated irrigation technology, and develops an intelligent CO_2_ injection system to inject CO_2_ through the roots. In this way, CO_2_ gas is transported to the vicinity of the plant roots through existing underground cavity tank drip irrigation pipes and dissipates into the enclosed environment. In this test, the 10-year-old ‘Flame Seedless’ grapes were used as the material, and the intelligent CO_2_ gas injection system was used for CO_2_ enrichment treatment. Then, we explored the effect of different CO_2_ concentrations on the photosynthetic characteristics and yield of the facility ‘Flame Seedless’ grapes, and the optimum CO_2_ concentration in grapes was picked out to achieve the optimal production effect, thus providing a theoretical basis for the application of aerated irrigation technology under controllable conditions of facility grapes in the future.

## 2 Materials and methods

### 2.1 Experimental site

The test was conducted in the sunlight greenhouse of Xinjiang Shihezi University Comprehensive Experimental Base (45°20′N, 86°03′E) from June 2022 to December 2023. The soil texture in the greenhouse was sandy soil, and the basic physical and chemical properties of the soil were as follows: pH 7.21, organic matter 13.67 g·kg^−1^, total nitrogen 0.38 g·kg^−1^, rapidly available phosphorus 25.6 mg·kg^−1^, and rapidly available potassium 21.3 mg·kg^−1^. The grape varieties in the greenhouse were ‘Flame Seedless’ table grapes planted in 2018. Vertical trellises were arranged with the trellis surface located east of the grape trees and in a north–south row direction. Cement upright pillars were erected at two ends of each grape tree row. Four galvanized iron wires were laid on the pillars, and the trellis was approximately 1.5 m. Five grape trees were planted in each row with a row spacing of 2.5 m and plant spacing of 0.8 m. Six fertile branches were retained on each grape tree during spring pruning. All grape trees in the greenhouse were irrigated and fertilized by an underground cavity tank drip irrigation system.

### 2.2 Experimental design

**Figure 1:**
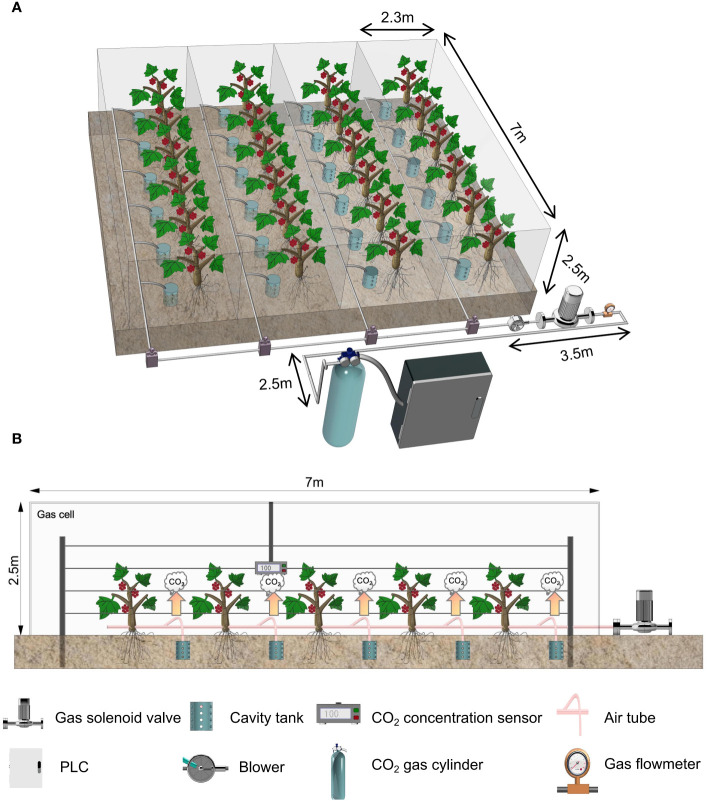
Schematic diagram of the test treatment. **(A)** CO_2_ gas injection system, which consists of a central control node (industrial computer and communication port), a gas application node (gas solenoid valve, gas electromagnetic flow meter, and convection device). **(B)** Closed artificial climate chamber, monitoring node: CO_2_ concentration sensor, gas application node: gas solenoid valve.

Four rows of grape vines with the same consistent growth in the greenhouse were selected for the test. Enclosed artificial chambers (2.5 m × 7 m × 2.3 m) made of polyethylene (PE) film were used to separate the rows of grape trees (**Figure 1**). Each chamber corresponded to a treatment, each treatment contained five grape vines, and each grape vine was regarded as a repeat. The gas exchange did not exist between each chamber and the external environment. Cylindrical complex polyvinyl chloride (PVC) cavities with a diameter of 10 cm and a height of 10 cm were buried in circular soil pits with a depth of 25 cm. Each soil pit was located 5 cm south of the grape vines. The bottom of the cavity tank was open, and small holes with a diameter of 1.5 cm were evenly distributed on the cavity tank body. Only a gas injection pipe with a diameter of 6 mm was connected to the top seal. The gas injection was performed in 35 days, from 25 June 2023 to 30 July 2023. Using a liquefied CO_2_ cylinder as the gas source, we injected CO_2_ with concentrations of (500 ± 30), (700 ± 30), (850 ± 30), and (1,000 ± 30) µmol·mol^−1^ and recorded these concentrations as 500 ppm, 700 ppm, 850 ppm, and 1,000 ppm, respectively. Root CO_2_ injection was adopted for each chamber. Specifically, after being released from the gas source, CO_2_ flows into the cavity tank in the vicinity of grape tree roots under the control of the CO_2_ injection control system and then dissipates to the entire chamber through small holes in the cavity tank. Different CO_2_ concentration levels were maintained in each chamber from 9:00 to 14:00 and from 17:00 to 22:00 each day, and the gas injection was stopped when the CO_2_ concentration in the gas chamber reached the processing setpoint. The CO_2_ concentration in each chamber was monitored using four CO_2_ concentration sensors suspended at a height of 0.5 m (**Figure 1**). The external surface of the chamber was washed with clear water every 3 days to prevent the light transmittance of films from being degraded by the accumulation of dust and sand in the air. We have collected the sunlight intensity, air temperature, and CO_2_ concentration in each chamber daily and recorded the data every 10 min since 25 June 2023.

### 2.3 CO_2_ injection system

The CO_2_ gas injection system consisted of a central control node, a monitoring node, and a gas application node (**Figure 1**). The monitoring node consists of a power supply module and a CO_2_ concentration monitoring module to collect the environmental information of each chamber. It obtains the CO_2_ concentration data in each chamber and transmits the environmental parameters to the central control node as current signals through wires. The gas application node comprises a power supply module, a gas solenoid valve, convection devices, and a gas electromagnetic flow meter. It mainly completes the execution of terminal commands and the feedback of control information. It also obtained control instructions analyzed from the terminal commands to determine the opening and closing of the gas solenoid valve in each chamber. Furthermore, the gas electromagnetic flow meter was used to monitor the real-time flow and transmit signals to the central control node to maintain the system’s stable operation. The central control node consists of a power supply module, industrial computer development module, and serial communication module to interact with chamber environment information and model processing. This node implements model processing through the industrial computer capable of human–computer interaction, combines the feedback information of the gas electromagnetic flow meter, and then sends a control signal to the gas application node. The central control node is responsible for starting up automatic equipment. It is located far from the north side of the chamber to prevent any human factor from affecting the test. The CO_2_ monitoring device is arranged in the middle of the chamber because this location can reflect the average CO_2_ concentration. The supplementary gas application node is placed near the central control and node, diverting the gas into the chamber through a gas duct. Grape trees are high, and CO_2_ has a larger relative molecular mass than air. After considering these factors, we set convection devices in the system chamber to reduce the gradient of CO_2_ concentration increase and ensure a uniform application of CO_2_. We could maintain the CO_2_ concentration in each chamber within the desired range depending on the information interaction between the nodes.

### 2.4 Sampling and measurements

#### 2.4.1 Leaf photosynthetic pigment content

Samples were collected on the 1st, 4th, 7th, and 10th day in the same irrigation cycle (21 July 2023 to 30 July 2023) after 25 days of treatment. Five repeated samples were set for each treatment, and three branches with similar consistent growth were randomly selected for each repeated sample. The well-grown functional leaves were selected and collected from basal node 3 to node 5 and immediately stored in a sealed bag. Then, the samples were taken back to the laboratory using a foam box with ice bags and tested to determine the content of chlorophyll a (Chl a), chlorophyll b (Chl b), and carotenoid in the grape leaves through spectrophotometry, and this was repeated five times. The specific methods were as follows: weigh 0.1 g of freshly washed leaves in a 5-mL centrifuge tube, add 2.5 mL of anhydrous ethanol and 2.5 mL of acetone, and leach the leaves overnight in the dark until the leaves turned white. The extract was poured into a 1-cm aperture cuvette, and the absorbance at 663 nm, 646 nm, and 470 nm was determined by zeroing with an extract reagent blank. Equations (1–4)


(1) $$\text{Chlorophyll a content }\left(\text{mg}\cdot {\text{g}}^{-1}\right)=12.21\times {\text{OD}}_{663}-2.81\times {\text{OD}}_{646}$$

(2) $$\text{Chlorophyll b content }\left(\text{mg}\cdot {\text{g}}^{-1}\right)=20.13\times {\text{OD}}_{646}-5.03\times {\text{OD}}_{663}$$

(3) $$\text{Carotenoid content }\left(\text{mg}\cdot {\text{g}}^{-1}\right)=\left(1,000{\text{OD}}_{470}-3.27\times \text{Chlorophyll a content}-104\times \text{Chlorophyll b content}\right)/229$$

(4) Chlorophyll a/b=Chlorophyll a content/Chlorophyll b content.

#### 2.4.2 Photosynthetic characteristics

We conducted a 4-day measurement on the 1st, 4th, 7th, and 10th day (21 July 2023 to 30 July 2023) in the same irrigation cycle after 25 days of treatment to determine the parameters related to the photosynthetic characteristics of the sampled leaves using an LI-6800 portable photosynthetic apparatus (LI-COR, USA), and repeated five times. The days of measurement were during sunny and cloudless days. At approximately 11:00 a.m., three paper strips of similar length were randomly selected for each treatment, and the functional leaves with good growth from basal nodes 3 to 5 were labeled, to determine the Pn, transpiration rate (Tr), stomatal conductance (Gs), and water use efficiency (WUE) of these leaves. The leaf chamber parameters were set as follows: photosynthetically active radiation (PAR) 1,500 μmol·m^−2^·s^−1^, VPD 0.1 kPa, flow rate 500 μmol·s^−1^, CO_2_ concentration of reference chamber 400 μmol·mol^−1^, and leaf chamber temperature 30°C.

#### 2.4.3 Photosynthesis–CO_2_ response curve and characteristic parameters

The light response curve and the CO_2_ response curve were determined after 35 days of treatment, and three function leaves were randomly sampled for each treatment. Measurements were performed using the LI-6800 portable photosynthesis apparatus and repeated five times. In the light response curve measurement process, only the light intensity was changed, and the light intensity gradients were set to 1,800, 1,500, 1,200, 900, 600, 300, 200, 150, 100, 50, and 0 μmol·m^−2^·s^−1^. When the light intensity changed, the minimum stable time was 120 s, and the maximum tough time was 200 s. The temperature was set at 30°C, the relative humidity was set at 50%, and the CO_2_ concentration was set at 400 μmol·mol^−1^. The measured data were used to simulate the light response curve and finally obtain the LSP, LCP, *P*_nmax_, and AQY. While calculating the CO_2_ response curve, we only changed the CO_2_ concentration gradients of light intensity into 400, 300, 200, 100, 50, 400, 600, 800, 1,000, 1,200, and 1,500 µmol·mol^−1^. When the CO_2_ concentration changed, the minimum stable time was 120 s, and the maximum tough time was 180 s. During the measurement, only the CO_2_ concentration was changed, the temperature was set at 30°C, the relative humidity was set at approximately 60%, and the light intensity was 1,500 μmol·m^−2^·s^−1^. The CO_2_ response curve was simulated using the measured data to obtain the maximum carboxylation rate (*V*_cmax_), the maximum electron transfer efficiency (*J*_max_), CO_2_ compensation point (CCP), and other related parameters.

#### 2.4.4 Leaf antioxidant enzyme and RuBisCO enzyme activities

Grape leaf samples were collected on the 35th day after treatment with different CO_2_ concentrations. For each treatment, five grape vine branches with similar growth were randomly selected, and the fourth to sixth functional leaves were collected from the base upward. Then, the samples were immediately transported back to the laboratory in a foam box with ice bags, washed with deionized water, and cut into blocks to measure the relevant enzyme activities, and this was repeated five times.

Antioxidant enzymes were determined by the method of Cakmak and Marschner (1992) using the azurotetrazole photochemical reduction method for superoxide dismutase (SOD), the guaiacol method for peroxidase (POD), the spectrophotometric method for catalase (CAT), and the catechol method for polyphenol oxidase (PPO). The soluble protein content was determined using the Caulmers Brilliant Blue G-250 method (Zou et al., 1995).

RuBisCO enzyme activity was determined by referring to the method of Reid et al. (1997) and using a plant enzyme-linked immunoassay kit (Suzhou Comin Biotechnology Co., China).

#### 2.4.5 Fruit yield

After the grapefruit reached the maturity stage (30 July 2023), three grape vines were randomly selected for each treatment. All fruit clusters on these grape vines were collected to determine the weight of every ear and calculate the grape production per grape vine. Three fruit clusters were randomly selected and a balance with an accuracy of 0.01 measured the weight of a single cluster. The final yield was obtained after conversion with the single cluster weight and plot area. Ten grapes were randomly picked from the upper, middle, and lower parts of a grape fruit cluster to determine the weight of a single grape, and this was repeated five times.

### 2.5 Data analysis

One-way analysis of variance (ANOVA) was performed using SPSS statistical software (version 22.0, IBM Electronics) to reveal the response of the measured variables to different CO_2_ concentrations. Duncan’s test and Tukey’s multiple comparison test using SPSS 20.0 (SPSS Inc., Chicago, IL, United States) were used to find significant differences between treatments (*P* < 0.05). Measurements were expressed as the mean ± standard error, and correlation analysis was performed using Pearson’s method. Graphing was performed using the Origin 2022 (Origin Software, Inc. Guangzhou, China) software.

## 3 Results

### 3.1 Effect of different CO_2_ concentrations on the content of photosynthetic pigments in the leaves of ‘Flame Seedless’ grapes

As shown in **Table 1**, the content of Chl b in the leaves treated with 700 ppm was significantly higher than in those treated with 500 ppm, 850 ppm, and 1,000 ppm of CO_2_ concentrations 1 day, 4 days, 7 days, and 10 days after irrigation (*P* < 0.05). Both Chl a and Chl b in the leaves 4 days, 7 days, and 10 days after irrigation showed 700 ppm > 850 ppm > 500 ppm > 1,000 ppm. The content of Chl b in the leaves treated with 700 ppm was significantly increased by 37.9%, 15.7%, and 30.0%, respectively, 4 days, 7 days, and 10 days after irrigation compared with that in the leaves treated with 500 ppm (*P* < 0.05). One day, 4 days, 7 days, and 10 days after irrigation, the content of carotenoids in the leaves treated with different concentrations of CO_2_ showed the following sequence: 700 ppm > 850 ppm > 1,000 ppm > 500 ppm. Chl a/b showed a trend of decreasing first and then increasing with the increase of CO_2_ concentration 1 day, 4 days, 7 days, and 10 days after irrigation. After 4 days and 10 days of irrigation, all treatments on Chl a/b showed 700 ppm < 850 ppm < 500 ppm < 1,000 ppm.

**Table 1:** Effect of different CO_2_ concentrations on the content of photosynthetic pigments in the leaves of ‘Flame Seedless’ grapes.

Days after irrigation	Treatment	Chl. a (mg·g^−1^)	Chl. b (mg·g^−1^)	Chl. a/b	Carotenoid (mg·g^−1^)
1	500 ppm	1.43 ± 0.05b	0.67 ± 0.00b	2.15 ± 0.12b	0.14 ± 0.02b
	700 ppm	1.57 ± 0.04a	0.73 ± 0.00a	2.15 ± 0.08b	0.37 ± 0.05a
	850 ppm	1.39 ± 0.04c	0.75 ± 0.05a	1.85 ± 0.18c	0.32 ± 0.03a
	1,000 ppm	1.35 ± 0.07c	0.59 ± 0.08c	2.28 ± 0.04a	0.20 ± 0.02b
4	500 ppm	1.73 ± 0.02b	0.87 ± 0.00c	1.99 ± 0.10b	0.19 ± 0.02c
	700 ppm	1.98 ± 0.12a	1.20 ± 0.04a	1.65 ± 0.17c	0.40 ± 0.09a
	850 ppm	1.80 ± 0.10b	0.99 ± 0.03b	1.81 ± 0.09b	0.38 ± 0.04a
	1,000 ppm	1.66 ± 0.03c	0.75 ± 0.02d	2.21 ± 0.09a	0.24 ± 0.02b
7	500 ppm	1.63 ± 0.07b	0.83 ± 0.01b	1.99 ± 0.12b	0.18 ± 0.01c
	700 ppm	1.88 ± 0.07a	0.96 ± 0.02a	1.92 ± 0.04b	0.39 ± 0.05a
	850 ppm	1.70 ± 0.01b	0.84 ± 0.02b	2.02 ± 0.07b	0.35 ± 0.03a
	1,000 ppm	1.56 ± 0.02c	0.72 ± 0.01c	2.17 ± 0.15a	0.23 ± 0.01b
10	500 ppm	1.64 ± 0.05b	0.70 ± 0.02b	2.34 ± 0.12b	0.16 ± 0.01c
	700 ppm	1.83 ± 0.09a	0.91 ± 0.04a	2.01 ± 0.12c	0.37 ± 0.04a
	850 ppm	1.65 ± 0.01b	0.72 ± 0.04b	2.29 ± 0.31b	0.37 ± 0.04a
	1,000 ppm	1.54 ± 0.01c	0.58 ± 0.00c	2.66 ± 0.05a	0.23 ± 0.01b

Data are the mean ± standard error (n = 3). Different letters indicate significant differences by Duncan’s test among treatments (P < 0.05). 500 ppm, CO_2_ concentration of 500 ± 30 µmol·mol^−1^; 700 ppm, CO_2_ concentration of 700 ± 30 µmol·mol^−1^; 850 ppm, CO_2_ concentration of 850 ± 30 µmol·mol^−1^; 1,000 ppm, CO_2_ concentration of 1,000 ± 30 µmol·mol^−1^.

### 3.2 Effect of different CO_2_ concentrations on the photosynthetic characteristics in the leaves of ‘Flame Seedless’ grapes

**Figure 2:**
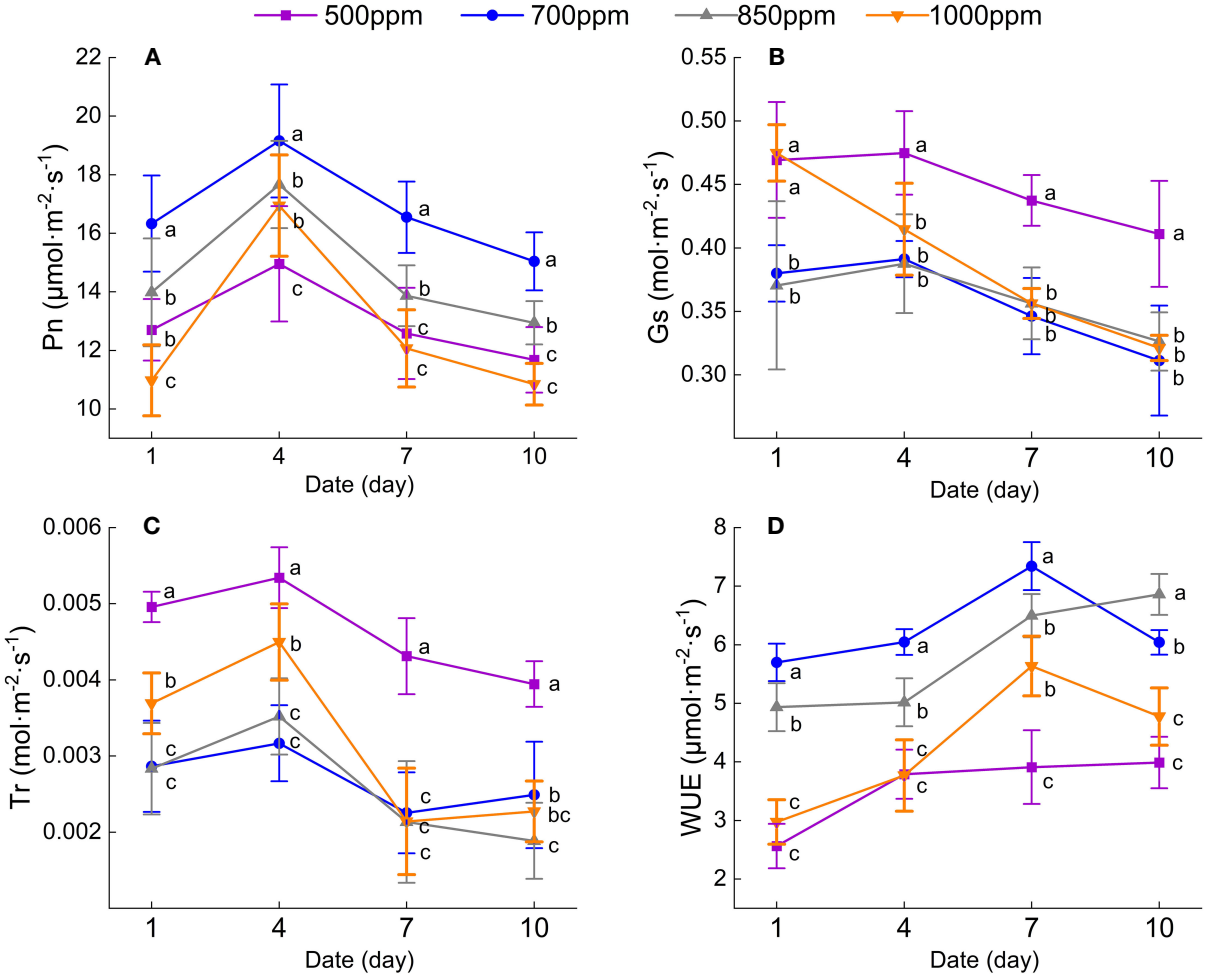
Effect of different CO_2_ concentrations on the photosynthetic characteristics of ‘Flame Seedless’ grape leaves. Data are the mean ± standard error (*n* = 3). Different letters indicate significant differences by Duncan’s test among treatments (*P* < 0.05). **(A)** Net photosynthetic rate of the leaves under different treatments. **(B)** Leaf stomatal conductance under different treatments. **(C)** Leaf transpiration rate under different treatments. **(D)** Leaf water use efficiency under different treatments. (1 d, 4 d, 7 d, 10 d) Different days after irrigation. 500 ppm, CO_2_ concentration of 500 ± 30 µmol·mol^−1^; 700 ppm, CO_2_ concentration of 700 ± 30 µmol·mol^−1^; 850 ppm, CO_2_ concentration of 850 ± 30 µmol·mol^−1^; 1,000 ppm, CO_2_ concentration of 1,000 ± 30 µmol·mol^−1^.

As shown in **Figure 2A**, the Pn of the leaves treated with different concentrations of CO_2_ shows a trend of increasing first and then decreasing in the same irrigation cycle. Four days, 7 days, and 10 days after irrigation, the Pn of the leaves in different treatments showed the following sequence: 700 ppm > 850 ppm > 500 ppm > 1,000 ppm. The Pn of the leaves treated with 700 ppm was significantly increased 28.6%, 28.1%, 31.6%, and 28.8%, respectively, 1 day, 4 days, 7 days, and 10 days after irrigation compared with that in the leaves treated with 500 ppm (*P* < 0.05). As can be seen from **Figures 2B, C**, both the Gs and Tr of the leaves in different treatments showed a trend of decreasing first and then increasing in the same irrigation cycle. Both the Gs and Tr of the leaves treated with 700 ppm were significantly lower than those of the leaves in other treatments and 10 days after irrigation (*P* < 0.05). Four days and 7 days after irrigation, both the Tr and Gs of the leaves in different treatments showed the following sequence: 500 ppm > 1,000 ppm > 850 ppm > 700 ppm. The leaves treated with 700 ppm were decreased by 42.2% and 40.7%, respectively, 1 day and 4 days after irrigation compared with that of the leaves treated with 500 ppm. The Tr of the leaves treated with 700 ppm was significantly decreased by 34.3% and 50.4%, respectively, 4 days and 10 days after irrigation compared with the leaves treated with 500 ppm (*P* < 0.05). As shown in **Figure 2D**, the grape leaves’ WUE in different treatments showed the same change trend with the Pn. The WUE of the leaves treated with 700 ppm was significantly higher than those treated with 500 ppm, 850 ppm, and 1,000 ppm 1 day, 4 days, 7 days, and 10 days after irrigation (*P* < 0.05). The WUE of the leaves treated with 700 ppm was increased by 122.4%, 116.2%, 151.7%, and 104.1%, respectively, 1 day, 4 days, 7 days, and 10 days after irrigation compared with that of the leaves treated with 500 ppm (*P* < 0.05).

### 3.3 Effect of different CO_2_ concentrations on the light response curve and characteristic parameters of ‘Flame Seedless’ grape leaves

**Figure 3:**
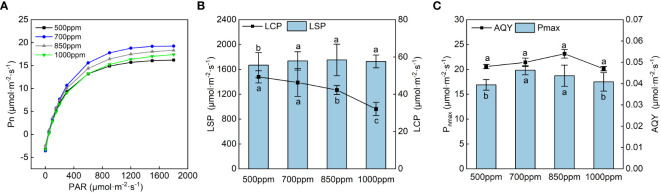
Effects of different CO_2_ concentrations on the light response curve and its characteristic parameters of grapevine leaves. Data are the mean ± standard error (*n* = 3). Different letters indicate significant differences by Duncan’s test among treatments (*P* < 0.05). **(A)** 500 ppm, CO_2_ concentration of 500 ± 30 µmol·mol^−1^; 700 ppm, CO_2_ concentration of 700 ± 30 µmol·mol^−1^; 850 ppm, CO_2_ concentration of 850 ± 30 µmol·mol^−1^; 1,000 ppm, CO_2_ concentration of 1,000 ± 30 µmol·mol^−1^. **(B)** LSP, light saturation point; LCP, light compensation point. **(C)** *P*_nmax_, maximum net photosynthetic rate; AQY, apparent quantum yield.

The light response curve of ‘Flame Seedless’ grape leaves in the color transformation period (**Figure 3A**) was obtained by fitting with the rectangular hyperbola correction model (Ye, 2010). Under the treatment of different CO_2_ concentrations, photosynthetically active radiation (PAR) significantly affected the Pn of the leaves. When the PAR was lower than 800 µmol·m^−2^·s^−1^, the Pn of the leaves in different treatments increased rapidly with the increase of PAR. When the PAR was higher than 1,000 µmol·m^−2^·s^−1^, the Pn of the leaves in other treatments increased slowly with the growth of the PAR. After the PAR exceeded 1,600 µmol·m^−2^·s^−1^, the Pn of the leaves treated with 700 ppm had the lowest increase rate compared with the leaves in other treatments. We calculated the LSP, LCP, *P*_nmax_, and AQY of ‘Flame Seedless’ grape leaves as per the light response curve fitting formula.

As shown in **Figure 3B**, the LCP showed a trend of gradually decreasing with the increase in CO_2_ concentration, while the LSP, *P*_nmax_, and AQY showed a trend of increasing first and then decreasing with the rise in CO_2_ concentration. In all the treatments, the LSP of the leaves showed 850 ppm > 700 ppm > 1,000 ppm > 500 ppm. The LSPs of the leaves treated with 700 ppm, 850 ppm, and 1,000 ppm were significantly increased by 4.06%, 4.98%, and 3.39%, respectively, compared with those treated with 500 ppm (*P* < 0.05). Under different CO_2_ concentrations, the leaves treated with 700 ppm had the highest *P*_nmax_, followed by those treated with 850 ppm. The AQY of the leaves in other treatments showed the sequence of 850 ppm > 700 ppm > 500 ppm > 1,000 ppm.

### 3.4 Effect of different CO_2_ concentrations on the CO_2_ response curve and characteristic parameters of ‘Flame Seedless’ grape leaves

**Figure 4:**
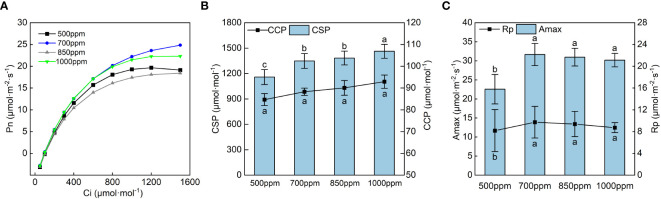
Effects of different CO_2_ concentrations on the CO_2_ response curve and its characteristic parameters of grapevine leaves. Data are the mean ± standard error (*n* = 3). In the same color, different letters indicate significant differences by Duncan’s test among treatments (*P* < 0.05). **(A)** 500 ppm, CO_2_ concentration of 500 ± 30 µmol·mol^−1^; 700 ppm, CO_2_ concentration of 700 ± 30 µmol·mol^−1^; 850 ppm, CO_2_ concentration of 850 ± 30 µmol·mol^−1^; 1,000 ppm, CO_2_ concentration of 1,000 ± 30 µmol·mol^−1^. **(B)** CSP, CO_2_ saturation point; CCP, CO_2_ compensation point. **(C)** *A*_max_, maximum photosynthetic capacity; *R*_p_, photorespiration rate.

The CO_2_ response curve of ‘Flame Seedless’ grape leaves in the color transformation period (**Figure 4A**) was obtained by fitting with the rectangular hyperbola correction model (Ye et al., 2010). The CO_2_ response curve reflected the law of Pn change of the grape leaves with the change of intercellular CO_2_ concentration (Ci). As shown in **Figure 4A**, the Pn of the leaves in different treatments increased rapidly when Ci was in the range of 0 to 600 µmol·m^−2^·s^−1^. When Ci was within the range of 600 to 1,200 µmol·m^−2^·s^−1^, the Pn of the leaves in different treatments increased at a lower rate with the increase of Ci. When Ci was within the range of 1,200 to 1,600 µmol·m^−2^·s^−1^, the Pn of the leaves treated with 500 ppm declined with the increase of Ci, and the Pn of the leaves in other treatments tended to be stable. We calculated the CSP, CCP, maximum photosynthetic capacity (*A*_max_), and photorespiration rate (*R*_p_) (**Figures 4B, C**) as per the CO_2_ response curve fitting formula.

**Figure 5:**
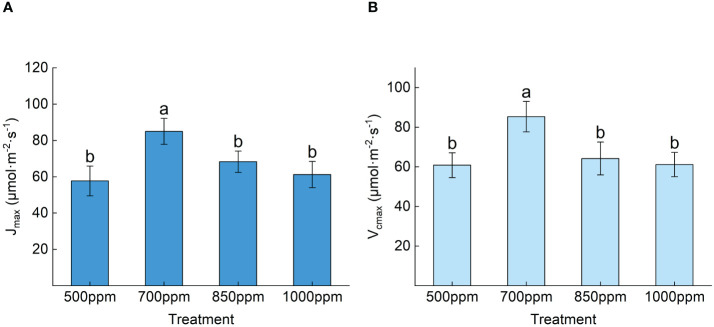
Effect of different CO_2_ concentrations on the maximum carboxylation rate and maximum electron transfer rate of ‘Flame Seedless’ grape leaves. Data are the mean ± standard error (*n* = 3). Different letters indicate significant differences by Duncan’s test among treatments (*P* < 0.05). **(A)** Maximum electron transfer rate of blades under different treatments. **(B)** Maximum carboxylation efficiency of the leaves under different treatments. 500 ppm, CO_2_ concentration of 500 ± 30 µmol·mol^−1^; 700 ppm, CO_2_ concentration of 700 ± 30 µmol·mol^−1^; 850 ppm, CO_2_ concentration of 850 ± 30 µmol·mol^−1^; 1,000 ppm, CO_2_ concentration of 1,000 ± 30 µmol·mol^−1^.

As seen in **Figure 4B**, the CSP and CCP showed a trend of increasing gradually with the increase in CO_2_ concentration, and the *A*_max_ and *R*_p_ showed a trend of increasing first and then decreasing with the rise in CO_2_ concentration. CCP showed the sequence of 850 ppm > 700 ppm > 1,000 ppm > 500 ppm, and the *A*_max_ and *R*_p_ of the leaves showed the sequence of 700 ppm > 850 ppm > 1,000 ppm > 500 ppm **Figure 4C**. As shown in **Figure 5**, both the maximum carboxylation rate and the maximum electron transfer rate showed the same change trend with the *A*_max_. The leaves treated at 700 ppm had the highest maximum carboxylation and electron transfer rates, followed by those treated at 850 ppm. Both the maximum carboxylation rate and the maximum electron transfer rate of the leaves treated with 700 ppm significantly increased by 54.57% and 51.37% (*P* < 0.05), respectively, compared with those treated with 500 ppm.

### 3.5 Effect of different CO_2_ concentrations on the activity of related enzymes in the leaves of ‘Flame Seedless’ grapes

**Figure 6:**
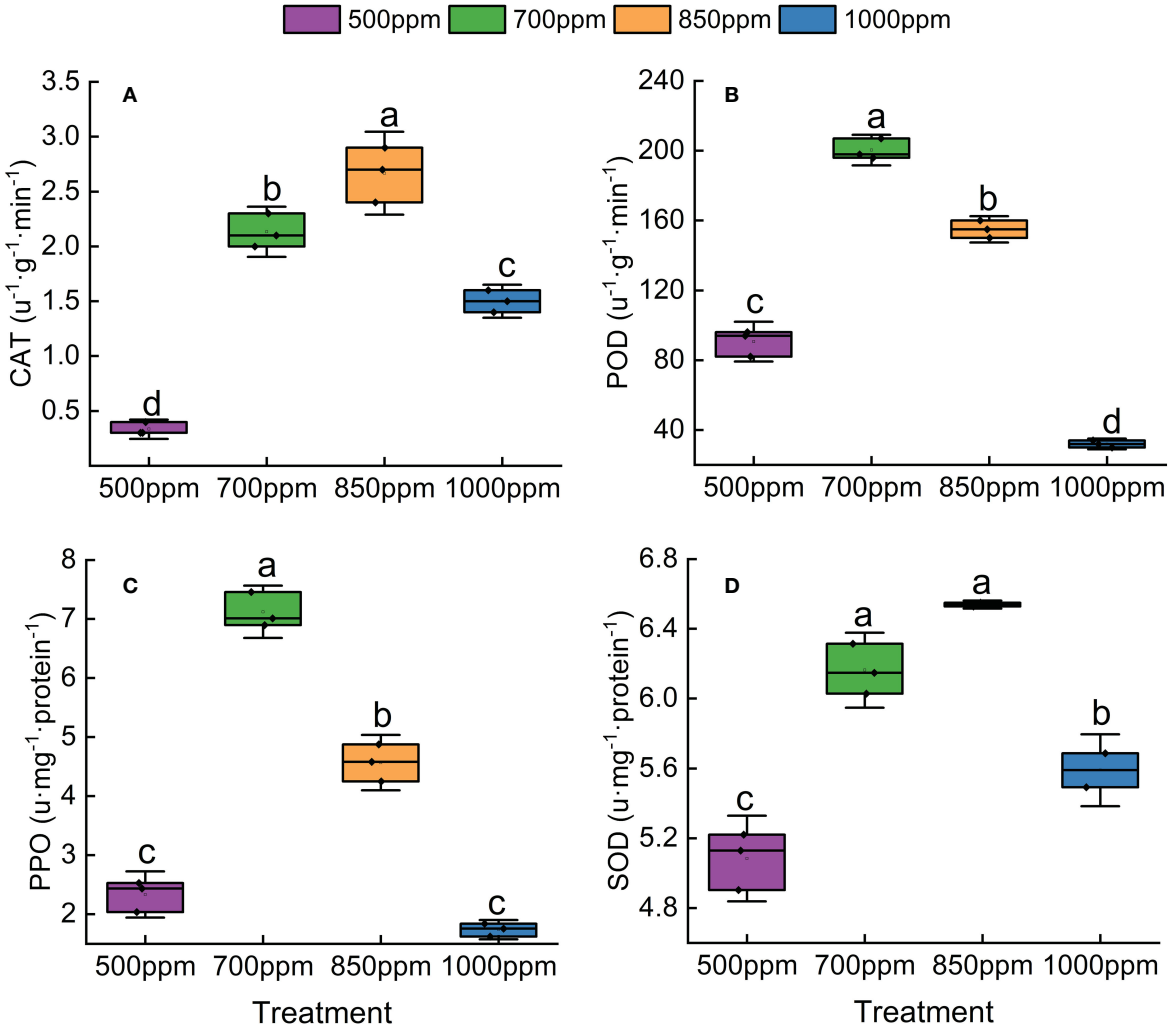
Effect of different CO_2_ concentrations on antioxidant enzymes in ‘Flame Seedless’ grape leaves. **(A)** Catalase activity under different treatments. **(B)** Peroxidase activity under different treatments. **(C)** Polyphenol oxidase activity under different treatments. **(D)** Superoxide dismutase activity under different treatments. Data are the mean ± standard error (*n* = 3). Different letters indicate significant differences by Duncan’s test among treatments (*P* < 0.05). 500 ppm, CO_2_ concentration of 500 ± 30 µmol·mol^−1^; 700 ppm, CO_2_ concentration of 700 ± 30 µmol·mol^−1^; 850 ppm, CO_2_ concentration of 850 ± 30 µmol·mol^−1^; 1,000 ppm, CO_2_ concentration of 1,000 ± 30 µmol·mol^−1^.

**Figure 7:**
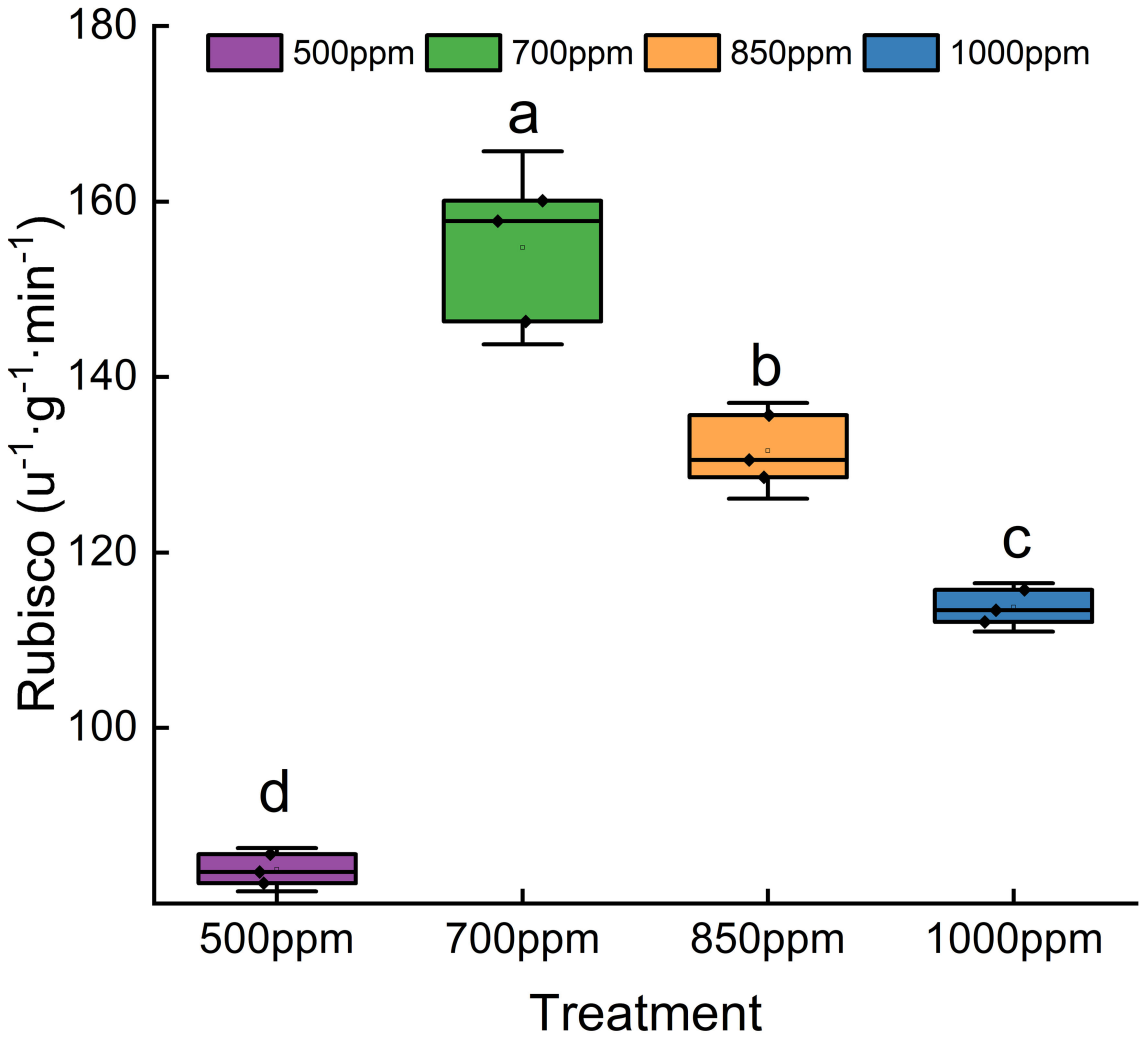
Effect of different CO_2_ concentrations on RuBisCO activity in ‘Flame Seedless’ grape leaves. Data are the mean ± standard error (*n* = 3). Different letters indicate significant differences by Duncan’s test among treatments (*P* < 0.05). 500 ppm, CO_2_ concentration of 500 ± 30 µmol·mol^−1^; 700 ppm, CO_2_ concentration of 700 ± 30 µmol·mol^−1^; 850 ppm, CO_2_ concentration of 850 ± 30 µmol·mol^−1^; 1,000 ppm, CO_2_ concentration of 1,000 ± 30 µmol·mol^−1^.

As shown in **Figure 6**, the activity of SOD, POD, CAT, and PPO showed a trend of increasing first and then decreasing with the increase of CO_2_ concentration. Both CAT and SOD in different treatments showed a sequence of 850 ppm > 700 ppm > 1,000 ppm > 500 ppm, while POD and PPO in different treatments showed a sequence of 700 ppm > 850 ppm > 500 ppm > 1,000 ppm. As shown in **Figure 7**, RuBisCO activity offered the same change trend with CAT, SOD, POD, and PPO with the increase of CO_2_ concentration. RuBisCO activity of the leaves treated with 700 ppm and 850 ppm was significantly increased by 89.11% and 66.12%, respectively, compared with that of the leaves treated with 500 ppm (*P* < 0.05) (**Figure 7**).

### 3.6 Effect of different CO_2_ concentrations on the yield of ‘Flame Seedless’ grapes

As shown in **Table 2**, the single grape mass, mass per fruit cluster, and yield of ‘Flame Seedless’ grapes treated with different concentrations of CO_2_ showed the sequence of 700 ppm > 850 ppm > 1,000 ppm > 500 ppm. The single grape mass of the grapes treated with 700 ppm, 850 ppm, and 1,000 ppm significantly increased by 37.9%, 26.8%, and 21.8%, respectively, compared with those treated with 500 ppm (*P* < 0.05). The yield of the grapes treated with 700 ppm and 850 ppm was 14.54 t·hm^−2^ and 12.91 t·hm^−2^, respectively, 3.04 t·hm^−2^ and 1.41 t·hm^−2^ higher than the yield of the grapes treated with 500 ppm.

**Table 2:** Effect of different CO_2_ concentrations on the yield components and yield of ‘Flame Seedless’ grapes.

Treatment	Quality of single grapes (g)	Mass per fruit cluster (g)	Yield (t·hm^−2^)
500 ppm	2.80 ± 0.05c	573.25 ± 9.30c	11.50 ± 2.13c
700 ppm	3.86 ± 0.13a	694.02 ± 12.56a	14.54 ± 2.73a
850 ppm	3.55 ± 0.03b	639.48 ± 5.63b	12.91 ± 1.14b
1,000 ppm	3.41 ± 0.04b	613.32 ± 6.70b	12.03 ± 1.31b

Data are the mean ± standard error (n = 3). Different letters indicate significant differences by Duncan’s test among treatments (P < 0.05). 500 ppm, CO_2_ concentration of 500 ± 30 µmol·mol^−1^; 700 ppm, CO_2_ concentration of 700 ± 30 µmol·mol^−1^; 850 ppm, CO_2_ concentration of 850 ± 30 µmol·mol^−1^; 1,000 ppm, CO_2_ concentration of 1^−^000 ± 30 µmol·mol^−1^.

### 3.7 Correlation of leaf photosynthetic pigment content, photosynthetic characteristics, photosynthetic CO_2_ curve characteristic parameters, and related enzyme activities with fruit yield

**Figure 8:**
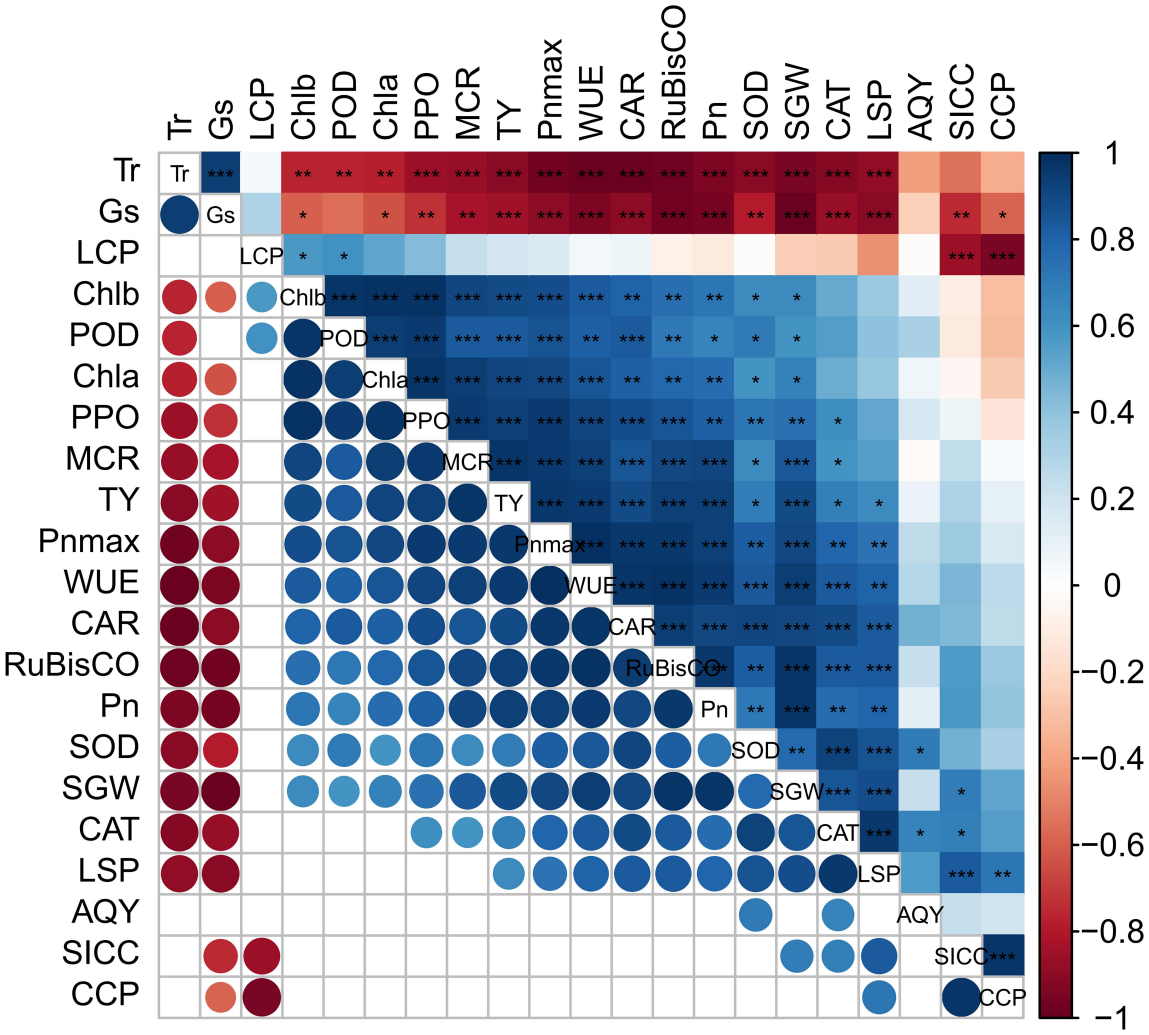
Correlation of leaf photosynthetic pigment content, photosynthetic characteristics, photosynthetic CO_2_ curve characteristic parameters, and related enzyme activities with fruit yield. Blue and red indicate significant positive and negative correlations, while white indicates no significant correlation. *: *P* ≤ 0.05; **: *P* ≤ 0.01; ***: *P* ≤ 0.001. CAR, carotenoid content in the leaves; CCP, CO_2_ compensation point; Pn, net photosynthetic rate; LCP, light compensation point; SICC, soil inorganic carbon content; SGW, single grape weight; LSP, light saturation point; MCR, maximum carboxylation rate of the leaves; Tr, transpiration rate; Gs, stomatal conductance; WUE, water use efficiency; AQY, apparent quantum yield; POD, peroxidase; CAT, catalase; SOD, superoxide dismutase; PPO, polyphenol oxidase; *P*_nmax_, maximum net photosynthetic rate; Chl a, chlorophyll a content; Chl b, chlorophyll b content; TY, total yield; RuBisCO: RuBisCO activity.

As shown in **Figure 8**, the SGW of ‘Flame Seedless’ grapes showed a highly significant positive correlation with the LSP, *V*_cmax_, Pn, WUE, and RuBisCO activity (*P* ≤ 0.01) and offered a significant positive correlation with Chl a content and Chl b content (*P* ≤ 0.05). Fruit yield had a significant positive correlation with chlorophyll content, Chl b content, carotenoid content, LSP, Pn, WUE, *V*_cmax_, CAT, POD, and RuBisCO activity (*P* ≤ 0.01) and a significant negative correlation with the Gs and Tr (*P* ≤ 0.01). In addition, the yield and SGW showed a significant negative correlation with carotenoid content (*P* < 0.05). Chlorophyll a and chlorophyll b contents significantly correlated with the CCP, Pn, Gs, and RuBisCO activity (*P* ≤ 0.05).

## 4 Discussion

### 4.1 Effect of different CO_2_ concentrations on the content of photosynthetic pigments in the grapes in the same irrigation cycle

Photosynthetic pigments play a basic role in the photosynthesis of plants. There are two kinds of chlorophyll, namely, Chl a and Chl b, which functions to capture, transfer, and convert light energy (Palit et al., 2020). A large number of studies demonstrated that an increased application of CO_2_ helped increase the chlorophyll content in the leaves of tomatoes, rice, wheat, and corn (Wang et al., 2013; Mamatha et al., 2014; Ksiksi et al., 2018; Mao et al., 2021). According to the study by Ullah et al. (2021), high CO_2_ concentration could effectively increase chlorophyll a and chlorophyll b in wheat leaves at the filling stage of wheat (Ullah et al., 2021). The results of this study showed that the chlorophyll a and chlorophyll b contents in the leaves were higher than 500 ppm when the CO_2_ concentrations were 700 ppm and 850 ppm, respectively. However, according to a study related to cherries, the content of chlorophyll a and chlorophyll b in cherry trees was much lower than 700 ppm when the CO_2_ concentration was 1,400 ppm (Druta, 2001). Feng et al. (2022) discovered that chlorophyll b content in *Schima superba* seedlings was lower than 400 ppm when the CO_2_ concentration was 1,000 ppm. We also obtained similar results based on this study. When the CO_2_ concentration was 1,000 ppm, the content of chlorophyll a and chlorophyll b in the leaves was lower than that under other CO_2_ concentrations. This situation implied that a high CO_2_ concentration could reduce the chlorophyll content in ‘Flame Seedless’ grape leaves because a high concentration of CO_2_ would promote the rapid growth of plants, thus causing a dilution effect and reducing the chlorophyll content. Based on this study, we also discovered that the content of photosynthetic pigments in the leaves treated with 700 ppm and 850 ppm would decrease in a smaller amplitude with the increase of days after irrigation. The study of Han et al. (2023) showed that the content of photosynthetic pigments of American fringe trees gradually decreased with the deepening of soil water stress. The finding implied that the treatments with 700 ppm and 850 ppm under experimental conditions could effectively moderate the effect of drought and other adverse conditions on plant photosynthetic pigments. Based on a study, Kant et al. (2012) found that high CO_2_ concentrations led to a decrease in crop chlorophyll a/b. In this study, CO_2_ at concentrations of 700 ppm and 850 ppm reduced the content of chlorophyll a/b. In comparison, a high concentration of CO_2_ (1,000 ppm) increased the content of chlorophyll a/b probably because the high concentration of CO_2_ weakened the promotive effect for chlorophyll b. Known as the auxiliary pigment in photosynthetic pigments, carotenoids could transfer the absorbed light energy to chlorophyll (Ashikhmin et al., 2023). Wang et al. (2015) found that an appropriately high concentration of CO_2_ could promote the rapid increase of carotenoid content in C_3_ plants, while an abnormally high concentration of CO_2_ (3,000 ppm) inhibited the promotion effect. Bao et al., 2016 also found that high concentrations of CO_2_ increased the growth rate of plants and inhibited the increase of carotenoid content. In this experiment, when the concentration of CO_2_ was 1,000 ppm, the increase of carotenoid content in the leaves was less than 700 ppm and 850 ppm, which shows that a too high CO_2_ concentration would also inhibit the efficiency of carotenoid content in the leaves of ‘Flame Seedless’ grapes.

### 4.2 Effect of different CO_2_ concentrations on the photosynthetic characteristics of grape leaves in the same irrigation cycle

Photosynthesis plays a critical role in the growth of plants (Cheng et al., 2023). Plant photosynthesis uses CO_2_ as a substrate for reactions, and the photosynthetic capacity of plants increases with the increase in CO_2_ concentration (Sanchez-Lucas et al., 2023). Previous studies have found that in a specific range of atmospheric CO_2_ concentration, high concentrations of CO_2_ could promote the Pn and WUE of the plant leaves (Khamis et al., 2023). In this study, the Pn and WUE of the leaves treated with 700 ppm and 850 ppm of CO_2_ were significantly higher than those of the leaves treated with 1,000 ppm, indicating that an abnormally high CO_2_ concentration would inhibit the photosynthesis of the leaves. The possible reason was that an unusually high CO_2_ concentration, a relatively low O_2_ concentration, and anaerobic respiration of plants produced toxic effects of ethanol, lactic acid, and other substances, thus inhibiting photosynthesis. As demonstrated by the study of Scher et al. (2022), photosynthesis did not continue to increase with the increase of atmospheric CO_2_ concentration. Still, it could reflect the changes in stomatal response and transpiration rate. Some relevant studies suggest that the increase in CO_2_ concentration would reduce the stomatal conductance of plant leaves (Zhang et al., 2022b), and high CO_2_ concentration would reduce not only the stomatal conductance but also the transpiration rate of the leaves (Liang et al., 2023). Among the different CO_2_ concentrations used in this study, the leaves treated with 700 ppm had the lowest Gs and Tr but the highest Pn and WUE. A decrease in Gs and an increase of *A*_max_ due to the increase of CO_2_ concentration were identified as a significant cause of the increase in plant WUE. Furthermore, this study discovered that both Gs and Pn show a trend of gradual decrease with the increase of days after irrigation and have the lowest decreasing amplitude at the CO_2_ concentration of 700 ppm, indicating that the treatment with 700 ppm effectively mitigates the effect of drought and other stresses on plant photosynthetic characteristics.

### 4.3 Effect of different CO_2_ concentrations on the light response curve and characteristic parameters of plants

The utilization of light energy in plants plays a vital role in the whole process of plant growth and development (Lu et al., 2019), and the ability of the plant leaves to respond to light can be reflected by the change in the plant’s light response curve (Serôdio et al., 2022). We could calculate the LSP, LCP, *P*_nmax_, and AQY of grape leaves per the fitting formula. According to relevant previous studies, the LCP of hops gradually decreased, and the AQY gradually increased with the increase of CO_2_ concentration (Bauerle, 2021). The study of Song et al. (2023) concluded that high CO_2_ concentration can increase the LSP and *P*_nmax_ of flue-cured tobacco, which coincided with the findings of this study. With the increase in CO_2_ concentration, the LCP of grapes decreased gradually, indicating that a high CO_2_ concentration can improve the ability of plants to utilize weak light. Plant LSP reflects the tolerance of plants to solid light (Pinnamaneni et al., 2022), and *P*_nmax_ reflects the maximum photosynthetic potential of plants (Niu et al., 2023). In this study, both LSP and *P*_nmax_ showed the sequence of 700 ppm > 850 ppm > 1,000 ppm > 500 ppm, indicating that the photosynthetic potential and tolerance of plants to solid light were the strongest when the CO_2_ concentration was 700 ppm, followed by 850 ppm. The AQY reflected the photosynthetic capacity of plants under weak light (Zhang et al., 2022a). In this study, the AQY of plants at 850 ppm CO_2_ concentration was the highest. In other words, when the CO_2_ concentration is 850 ppm, the plant has the strongest adaptability to weak light. The results of this study showed that the appropriately high concentration of CO_2_ can significantly improve the photosynthetic curve characteristic parameters of ‘Flame Seedless’ grapes. Specifically, the CO_2_ concentration of 700 ppm and 850 ppm contributed to the optimal effect.

### 4.4 Effect of different CO_2_ concentrations on the CO_2_ response curve and characteristic parameters of plants

We can judge the level of demand for CO_2_ in the external environment according to the change in CO_2_ response characteristic parameters. The main photosynthetic curve characteristic parameters are the CSP, CCP, *A*_max_, *R*_p_, *V*_cmax_, and *J*_max_. *V*_cmax_ and *J*_max_ are two critical parameters that characterize the photosynthetic capacity of plants (Bermudez et al., 2021). The study of Fauset et al. (2019) showed that a high CO_2_ concentration significantly increases *V*_cmax_ and *J*_max_. According to the findings of this study, both *V*_cmax_ and *J*_max_ of the plants treated with 700 ppm and 850 ppm of CO_2_ were significantly higher than those of the plants treated with 500 ppm, probably because an appropriate increase of CO_2_ concentration has increased the substrate of photosynthesis and then promoted the rate of carboxylation and the rate of electron transfer in the plant. An appropriate increase in CO_2_ concentration can effectively promote the carboxylation rate and electron transfer rate of ‘Flame Seedless’ grapes. The study of Lamba et al. (2018) on *Picea* suggested that an abnormally high CO_2_ concentration would inhibit the increase of *V*_cmax_ and *J*_max_. We have obtained a similar result with the above study, indicating that *V*_cmax_ and *J*_max_ of the plants treated with 1,000 ppm were significantly lower than those of the plants treated with other concentrations of CO_2_. This may be due to the continuous increase in CO_2_ concentration, which accelerates the accumulation of plant biomass and reduces the N content in plant leaves, leading to an overall decrease in leaf protein content, resulting in a decrease in the quantity or activity of RuBisCO protein per unit leaf area of plants, ultimately limiting the carboxylation efficiency of RuBP (Andrews et al., 2019). In accordance with the study of Xiao et al. (2020), high CO_2_ concentration significantly increased the CSP, CCP, and *A*_max_ of plants and reduces the *R*_p_. In this study, the CO_2_ concentrations of 700 ppm and 850 ppm significantly increased the CSP, CCP, and *A*_max_ of ‘Flame Seedless’ grapes. However, the CO_2_ concentration of 1,000 ppm reduced the *A*_max_ of the grapes. The possible reason was that a high CO_2_ concentration reduces plant WUE and inhibits plant photosynthesis, thereby weakening the maximum photosynthetic capacity of the plants. When the CO_2_ concentration was 700 ppm, ‘Flame Seedless’ grapes have higher photosynthetic performance and stronger carbon fixation and carboxylation capacity.

### 4.5 Effect of different CO_2_ concentrations on the activity of antioxidant enzymes and RuBisCO in the leaves of grapes

CAT, POD, SOD, and PPO have been identified as four important protective enzymes in the plant antioxidant enzyme system (Sinan et al., 2020; Gouda et al., 2023). The relevant previous studies demonstrated that the activity of antioxidant enzymes in plants increases with the increase of CO_2_ concentration and then decreases sharply or gradually after reaching a peak value (Hu et al., 2020). In this study, the activity of CAT and SOD increased sharply with the increase of CO_2_ concentration when the CO_2_ concentration was in the range of 500 ppm to 850 ppm, reached the maximum when the CO_2_ concentration was 850 ppm, and decreased sharply since then. The activity of POD and PPO increased sharply with the increase of CO_2_ concentration when the CO_2_ concentration was in the range of 500 ppm to 700 ppm, reached the maximum when the CO_2_ concentration was 700 ppm, and decreased gradually since then. This conclusion coincides with the findings of the relevant previous studies (Zhang et al., 2020). It indicates that CO_2_ in a specific range of concentrations can effectively protect grape leaves from oxidative damage probably because the increase of CO_2_ concentration could enhance stomatal resistance, reduce transpiration rate, and increase the WUE, thereby resulting in a stronger tolerance of plants to stress. Moreover, more nicotinamide adenine dinucleotide phosphate (NADPH) was formed by the electron transfer system in the process of photosynthesis regulation with the increase of CO_2_ concentration, thus promoting the ascorbate–glutathione cycle and improving the activity of related antioxidant enzymes. Aydi et al. (2020) believed that the decrease in antioxidant enzyme activity was caused by the situation that CO_2_ enrichment reduced the demand of removing active oxygen in cellular metabolism. The decrease of antioxidant enzyme activity in the grapes treated with a high concentration of CO_2_ was probably caused by the situation that CO_2_ enrichment can increase the pCO_2_/O_2_ ratio and CO_2_ assimilation and reduce the formation of active oxygen with O_2_ as electron acceptors. However, the increase in CO_2_ concentration can reduce H_2_O_2_ formed by photorespiration. Furthermore, the increase in CO_2_ concentration may reduce the demand of cells for antioxidant activity. Plant photosynthetic performance was jointly affected by the leaf antioxidant system, RuBisCO activity, and other related factors. As demonstrated by the study of Sharwood et al. (2016), an appropriate CO_2_ concentration can promote RuBisCO carboxylation activity and then enhance plant photosynthesis. This study showed that RuBisCO activity increased rapidly when the CO_2_ concentration was 500 ppm to 700 ppm, reached the maximum when the CO_2_ concentration was 700 ppm, and decreased slowly since then. This situation suggests that an appropriate increase of CO_2_ activity can effectively promote RuBisCO activity of ‘Flame Seedless’ grape leaves but an abnormally high concentration would inhibit the initial promotive effect of CO_2_. The possible reason was that the distribution of nutritional elements in plant bodies was affected if the CO_2_ concentration was too high. As a result, RuBisCO synthesis was inhibited.

### 4.6 Effect of different CO_2_ concentrations on the yield of ‘Flame Seedless’ grapes and correlation between the indicators

CO_2_ concentration is one of the important environmental factors affecting photosynthesis. Increased CO_2_ concentration helps promote the photosynthetic reaction rate of plants and accelerate plant growth and yield accumulation (Yamaura et al., 2023). A large number of scholars have conducted extensive studies to explore the effect of increased atmospheric CO_2_ concentration on plant photosynthesis and yield and concluded that increased CO_2_ concentration could promote plant photosynthesis and yield (Kanno et al., 2017; Choi and Kang, 2019; Akhlaq et al., 2023). According to the results of this test, CO_2_ concentrations of 700 ppm and 850 ppm had significantly increased single grape weight and total yield. When the CO_2_ concentration was 700 ppm, the largest increasing amplitude occurred. The smallest increasing amplitude was when the CO_2_ concentration was 1,000 ppm. The yield of grapes treated with 700 ppm and 850 ppm of CO_2_ has been increased by 3.03 t·hm^−2^ and 1.41 t·hm^−2^, respectively, compared with those treated with 500 ppm. As reflected by the above data, the increasing amplitude of single grape weight and yield of the grapes treated with 1,000 ppm had decreased probably because an abnormally high CO_2_ concentration (1,000 ppm) will reduce the concentration of most mineral elements in plant bodies, thereby inhibiting plant yield. Many studies demonstrate that photosynthetic pigment content, photosynthetic characteristic parameters, and photosynthetic performance have a direct effect on the final yield of plants (Peng et al., 2020; Markovic et al., 2021; Zhang et al., 2023a). As reflected by the findings of this study, fruit yield showed a highly significant positive correlation with the Pn, WUE, photosynthetic pigment content, and RuBisCO activity and a significant positive correlation with the antioxidant enzyme activity of the leaves. The above correlations indicated that photosynthetic upregulation can effectively promote the formation of ‘Flame Seedless’ grape yield, and an enhanced antioxidant system can help avoid severe yield reductions of ‘Flame Seedless’ grapes. The RuBisCO activity of plant leaves mainly reflects the RuBP carboxylation rate. Any change in the activity will directly affect the carbon fixation and carboxylation capacity of the plants and further affect the formation of fruit yield (Tanambell et al., 2024). According to the correlation between RuBisCO activity and yield in this study, the increased RuBisCO activity could improve the carbon assimilation efficiency of ‘Flame Seedless’ grapes, thus promoting the formation of fruit yield.

## 5 Conclusion

In conclusion, CO_2_ concentrations of 700 ppm and 850 ppm significantly increased the photosynthetic pigment content and physiological characteristics of grape leaves, promoting an increase in grape yield. The photosynthetic pigment content was higher in CO_2_ concentrations of 700 ppm and 850 ppm than in other treatments, with leaf stomatal conductance and transpiration rate lower in CO_2_ concentration of 700 ppm than in other treatments. The light saturation point, CO_2_ saturation point, and maximum photosynthetic capacity are all at their maximum values when the CO_2_ concentration is 700 ppm. The antioxidant enzyme activity is higher than the other treatments, and the yield is the highest, at 14.54 t·hm^−2^. Compared to the control treatment, the yield increased by 3.04 t·hm^−2^. The promotion effect of plant photosynthesis and yield is better when the CO_2_ concentration is 700 ppm. When the CO_2_ concentration is 1,000 ppm, grape photosynthesis is inhibited. This study provides a certain theoretical basis for achieving carbon neutrality and green and sustainable development under the conditions of digital agriculture in future facility production. Due to limitations in research content, this paper only compares the aboveground photosynthetic performance and fruit yield. Further research is needed on the impact of different CO_2_ concentrations on the rhizosphere environment.

## Data availability statement

The original contributions presented in the study are included in the article/supplementary material. Further inquiries can be directed to the corresponding authors.

## Author contributions

YZ: Writing – original draft. HM: Writing – original draft. JX: Writing – review & editing. DY: Writing – review & editing. HZ: Writing – review & editing. XL: Writing – review & editing. KY: Writing – review & editing. FZ: Writing – review & editing.
